# TarGo: network based target gene selection system for human disease related mouse models

**DOI:** 10.1186/s42826-019-0023-z

**Published:** 2019-11-13

**Authors:** Daejin Hyung, Ann-Marie Mallon, Dong Soo Kyung, Soo Young Cho, Je Kyung Seong

**Affiliations:** 1 National Cancer Center, 323 Ilsan-ro, Goyang-si, Kyeonggi-do 10408 Republic of Korea; 2 MRC Harwell Institute, Mammalian Genetics Unit, Oxfordshire, OX11 0RD UK; 3 Laboratory of Developmental Biology and Genomics, Research Institute for Veterinary Science, and BK21 Plus Program for Creative Veterinary Science, College of Veterinary Medicine, Seoul National University, Seoul, 08826 Republic of Korea; 4 Korea Mouse Phenotyping Center (KMPC), Seoul National University, Seoul, 08826 Republic of Korea; 5 Interdisciplinary Program for Bioinformatics, Program for Cancer Biology and BIO-MAX institute, Seoul National University, Seoul, 08826 Republic of Korea

**Keywords:** Systems biology, Genetic engineered mice, Bioinformatics, PageRank algorithm, Database

## Abstract

Genetically engineered mouse models are used in high-throughput phenotyping screens to understand genotype-phenotype associations and their relevance to human diseases. However, not all mutant mouse lines with detectable phenotypes are associated with human diseases. Here, we propose the “Target gene selection system for Genetically engineered mouse models” (TarGo). Using a combination of human disease descriptions, network topology, and genotype-phenotype correlations, novel genes that are potentially related to human diseases are suggested. We constructed a gene interaction network using protein-protein interactions, molecular pathways, and co-expression data. Several repositories for human disease signatures were used to obtain information on human disease-related genes. We calculated disease- or phenotype-specific gene ranks using network topology and disease signatures. In conclusion, TarGo provides many novel features for gene function prediction.

## Introduction

In the post-genome era, the functional analysis of protein-coding genes remains an important goal and a major challenge for the field of biology. To find novel gene functions, various genetically engineered mouse (GEM) models have been used, involving the use of mutagens such as N-ethyl-N-nitrosourea (ENU), transposons, gene trapping, and gene targeting. Recently, the International Mouse Phenotyping Consortium (IMPC) started generating knockout mice for every mouse gene and collecting phenotyping data for each null mutation [1]. To accelerate this goal, a targeted gene selection system for GEM models was deemed necessary.

Because GEM constructions require a considerable amount of time and money, target gene selection is considered the most important step. To select GEM target genes using an unsupervised method, two different approaches were applied in systems biology. First, high throughput data analysis based approaches have been employed. GEM target genes are predicted using Omics data analysis or cross-species gene conservation [2–4]. Those approaches provide the opportunity to find novel gene functions and putative driver genes in disease, but are of limited use when predicting sample sensitivity. Second, ontology structure-based approaches have been employed. Researchers have considered the integration between human disease and mouse phenotype ontologies [5, 6]. The PhenomicDB and PhenoHM databases use text-matching approaches between two different terms [7, 8]. MouseFinder, PhenoDigm, and PhenomeNet databases provide gene-disease or gene-phenotype associations through cross-species phenotype comparisons [9–11]. Ontology-based approaches have a good prediction performance for gene function, but cannot predict the function of non-annotated genes. Recently, integration approaches between high throughput data and network search are proposed. ExomeWalker provides an integrated approach to rank candidate genes using a random-walk with restart algorithm [12]. Hwang and colleagues proposed a co-clustering method between phenotypes and genes [13]. The limitations of an integration method are the dependence on pre-constructed datasets and non-optimal filtering strategies for false positives. The goal of those previous approaches is gene function estimation, but limitations are remained.

Here, we propose an approach, called Target gene selection system for Genetically engineered mouse models (TarGo). We predicted mouse gene function with molecular interactions and sorting relationship for phenotype (or disease) signature genes. We calculated association between phenotype (or disease) signature gene and other gene using the Topic-Sensitive PageRank (TSPR) and the TrustRank algorithms [14]. The gene interaction network was constructed using PPIs, molecular pathway analysis, and HumanNet. The phenotype (or disease) signature genes are selected from MeSH, HPO, GWAS Central, and Orphanet [15–18]. We evaluated the resulting prioritized genes according to the known genotype-phenotype associations contained in Mouse Genome Informatics (MGI). Therefore, TarGo includes many novel features for gene function predictions. The Web server is available at http://combio.snu.ac.kr/targo.

## Results

### Construction of mouse network

The gene interaction network was composed of three molecular interaction databases; 377,473 PPIs from NCBI GeneRIF, 88,279 pathways from Pathway Commons, and 882,705 Co-expression data from HumanNet [19–21]. From herein, nodes will refer to genes and edges will refer to the genetic interaction between the genes. To predict human disease-related genes in the mouse model, a network was constructed using orthologous genes in humans and mice.

**Fig. 1:**
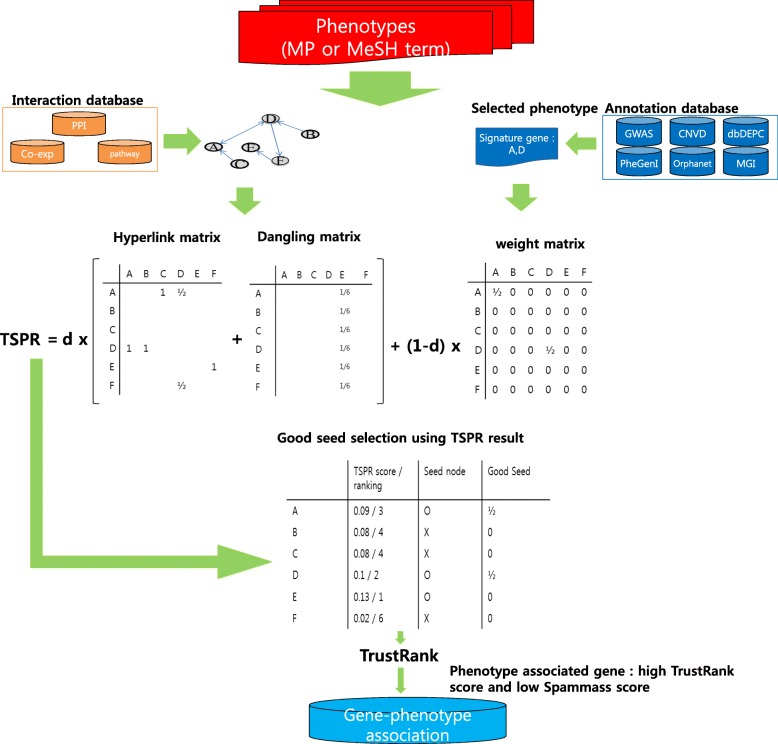
Prediction of gene-phenotype association using interaction network and signature genes. The hyperlink matrix and dangling matrix (no outgoing edge matrix) were constructed from interaction databases (red box). The weight matrix was constructed using public annotation databases (orange box). The d represented the dumping factor (0.85). The TSPR score represented the gene association for that particular phenotype. For this given TSPR score vector, A and D were signature genes. The seed nodes were selected from the top three ranking TSPR scores. A and D were good seeds because these two genes were signature genes in the input phenotype and ranked among the top three in the TSPR result. Therefore, d is 2 in this figure, meaning the good seed vector is ½. All other cases were given 0. Finally, phenotype-associated genes were selected from those with a high TrustRank score and low Spammass score

To select orthologous genes, we used NCBI’s Homologene (http://www.ncbi.nlm.nih.gov/homologene). In total, 16,353 orthologous genes and 1,204,365 interactions were selected from three repositories described above. Most (81%) of these genes and 10% of the interactions overlapped in all three databases. The TSPR algorithm required the hyperlink matrix, which is an n × n matrix for a given interaction network, where n is the total number of nodes in the network. All nodes were designated an “out-degree” value, which is the number of outgoing edges (interactions) for that specific node. If gene i had links to gene j, i would provide 1/out-degree to j in the hyperlink matrix (Fig. 1). If gene i did not have outgoing edges, then this gene was termed a “dangling node,” and all dangling nodes were collected in a dangling matrix. The dangling matrix was given the constant value 1/n.

### Construction of weight matrix from the annotation database

The weight matrix in the TarGo system was constructed using signature genes. If genes were not annotated to a specific phenotype, values in the weight matrix were 0. If genes were annotated to a specific phenotype (signature genes), values were 1/G (G is total number of signature genes for a specific phenotype). The weight matrix consisted of the number of nodes for each selected phenotype, which were used to generate data on genes (human and mouse) and their associated phenotypes. The information originated from multiple resources; GWAS information was collected from GWAS Central, PheGenI, and from public repositories for genetic association studies [15, 22]. CNV data were selected from the CNVD database, a comprehensive resource for CNVs and related diseases [23]. We obtained data on abnormal protein expression in human disease from dbDEPC, which provided an overview of protein level expression changes, mainly detected by mass spectrometry [24]. Finally, pre-annotation data was collected from MGI and Orphanet [18, 25]. In total, this resulted in 16,305 genes, 3330 phenotypes, and 326 diseases to populate the test database. The human disease terms were converted to MeSH or Orphanet ID. The human phenotypes were converted to Mammalian Phenotype (MP) term using HPO associations. MP term was constructed for annotating mouse knockouts, mutations, and other type alleles at the Mouse Genome Informatcs (MGI) database. MP ontology provides phenotype reference for the observable morphological and behavioral character.

### Hub node effect in ranking

A hub node in a network is defined as a highly connected node and will be predicted putative candidate with high frequency. Using the Pearson method, we examined the association between the TrustRank score and node degree. The correlation was calculated for each MP term or MeSH term. For 98% of MP terms, the correlations between node degree and TrustRank score were lower than 0.6, while 90% of MeSH terms had a correlation coefficient lower than 0.6. In most phenotypes, the hub node was not highly ranked (Additional file 1: Figure S1). We used the same network topology in the gene ranking calculation, and the association score was calculated to be dependent on other topics. This results indicates that hub node is not frequently predicted in network and putative candidates will be associated for signatures genes.

### Evaluation of a known phenotype

**Fig. 2:**
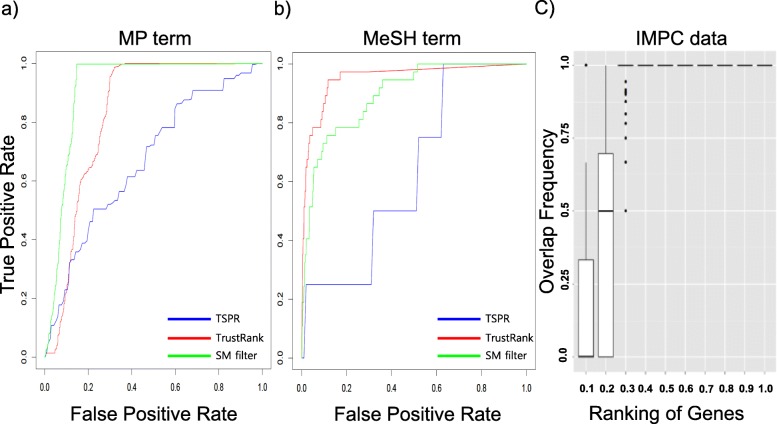
ROC curve for TarGo prediction results. **a** In the MP term, the AUC score of TSPR is 0.66, TrustRank is 0.82, and SM filter is 0.91. **b** In the MeSH term, the AUC score of TSPR is 0.63, TrustRank is 0.95, and SM filter is 0.93. **c** Overlapping between IMPC and TarGo. the X-axis indicates high-ranking genes sorted by rank score. The Y-axis is the overlapping percentage between IMPC and TarGo

To validate our method, we evaluated the prediction results according to known gene-disease and gene-phenotype associations. All the information for known gene-phenotype associations was collected from the “Mouse/Human Orthology with Phenotype Annotations” table in MGI. A known gene-disease model was established from the “Genotypes with Both Phenotype and Disease Annotations for Marker Type Genes, Excluding Conditional Mutations” and “Genotypes with Both Phenotype and Negated Disease Annotations for Marker Type Genes, Excluding Conditional Mutations” tables in MGI. We selected the top 100 genes from the prediction results. Receiver operating characteristic (ROC) analysis was performed using the R ROCR package [26]. When the TrustRank method was used in MP, the AUC score increased from 0.66 to 0.91 and performance was further increased using the Spammass filter (Fig. 2a). Using the TrustRank method in MeSH, the AUC score increased from 0.63 to 0.95 and slightly decreased to 0.93 by further using Spammass filter (Fig. 2b). We suggest that more genes are associated with disease terms (annotated with MeSH) than with phenotype terms; however, TarGo still exhibits good performance (AUC = 0.93). This result could be due to the complexity of the disease, as a disease will normally be related to various genes and phenotypes.

For a cutoff guideline, we compared our prediction results to IMPC mouse phenotypes validated by the high-throughput pipeline (Fig. 2c). From the IMPC database, 673 genes were selected (*P* < 0.0001). In the TarGo data, 3291 genes were selected. Because the TarGo prediction result depended on the amount of knowledge, we selected high-degree (or high annotation) genes, which comprised 2% of all genes in the network. The results showed that 101 genes over-lapped between IMPC and TarGo. The top 20% of the TarGo prediction results overlapped with > 70% of the IMPC results, and the top 30% of the TarGo prediction results overlapped with 100% of the IMPC results. Most TarGo-selected highly ranked genes considerably overlapped with IMPC and showed good performance.

### Web contents of TarGo

**Fig. 3:**
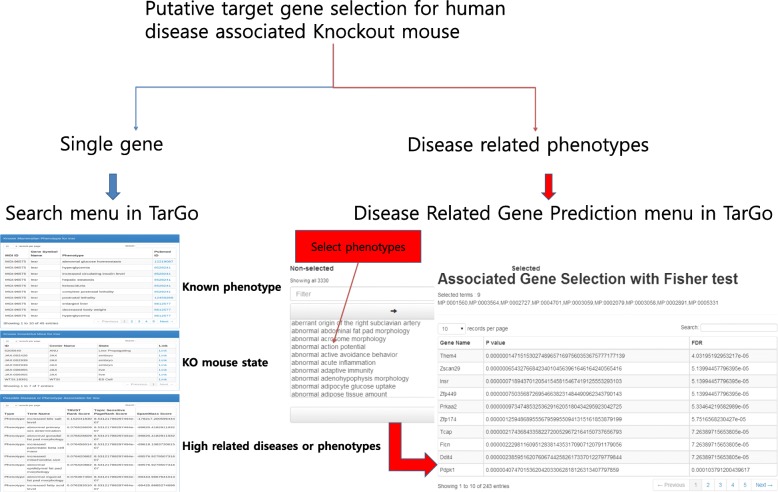
Contents of the TarGo database. **a** The user can find the knock out (KO) target gene using the TarGo search system. Known phenotype, KO mouse state, and high-related phenotype are provided using gene name (blue line). High-associated genes are provided using multiple phenotype selection (red line). **b** The user can analyze gene rank using in-house data. The researcher can also use various in-house data, including gene sets from the Omics approach or empirical knowledge. Therefore, the user can regenerate gene rank with user-defined gene sets or network (green line)

Association between gene and phenotype: Pre-computed genotype-disease and genotype-phenotype associations can be searched using the gene symbol, MeSH term, or MP term. Using the search menu, users can find prediction results as well as known phenotypes, such as known mammalian phenotypes, known GEM models, and possible phenotype associations (Fig. 3).

These novel phenotype candidates were confirmed using high-throughput mouse phenotyping data in IMPC. For example, knockout (KO) mouse phenotypes for the genes Ndfip2 and Spop were not annotated in MGI. We predicted novel phenotypes of significant changes in sodium levels in the Ndfip2 KO mouse and increased circulating potassium levels in the Spop KO mouse. In mouse phenotyping data, these phenotypes were evident as a significant change between normal and deficient mice (*P* = 0.0005 and 0.007, respectively). The Slc40a1 KO mouse displays abnormalities in embryogenesis, growth size, and the hematopoietic and immune systems [27]. TarGo predicted a novel phenotype and abnormal bone mineralization, which was identified by IMPC phenotype data (*P* = 0.0000005). The Pfn1 KO mouse phenotype is annotated association with adipose tissue, growth/size, homeostasis/metabolism, integument, limbs/digits/tail, and mortality/aging [28]. TarGo predicted a novel phenotype of abnormal iris morphology, and we confirmed this result in the mouse phenotype data (*P* = 0.009). Slc25a4-deficient mice show abnormal cardiovascular, homeostasis, and muscle phenotypes [29]. TarGo predicted several novel phenotypes for Slc25a4 KO, such as increased circulating calcium levels (MP:0000194), decreased circulating glycerol levels (MP:0003442) and increased heart weight (MP:0002833). We found some significant changes in IMPC phenotype data (*P* = 0.000007, 6e-16, respectively). Arpc1b KO mice was reported homeostasis phenotype (1). We found a novel function for decreased circulating serum albumin levels (MP:0005419) in TarGo and IMPC (*P* = 0.0001). Abnormalities in behavior, growth/size, hematopoiesis, homeostasis, immune system, integuments, and vision/eyes are known phenotypes for Clk1 KO mice [30]. A novel phenotype of an abnormal adipose tissue amount (MP:0005452) was predicted in TarGo, and significant phenotypes were confirmed in IMPC (*P* = 0.002).

GEM target gene prediction with selected mouse phenotypes: TarGo identifies highly ranked genes for user-selected phenotypes using the Fisher exact test in conjunction with the TrustRank algorithm, allowing the user to select the target gene for their GEM model (Fig. 3a).

We performed gene selection using this system by predicting diabetes and obesity-related genes in mice. Six MP terms for type 2 diabetes and obesity (MP:0005293 glucose intolerance, MP:0002079 hyperinsulinemia, MP:0001792 impaired wound healing, MP:0002628 liver steatosis, MP:0001433 hyperphagia, and MP:0001552 hypertriglyceridemia) were selected from the Jackson Laboratory (B6 ob and B6 db). Highly associated genes for the selected phenotypes were predicted by FDR < 0.01. Finally, six genes (Sec61a1, Insr, Sirt1, Pdpk1, Sin3a, and Sqstm1) were predicted to be associated with diabetes and obesity. Four of these genes have been used in diabetes and obesity studies. Sec61a1-defective mice have been used as a diabetes model [31]. Insr is a major risk factor for human obesity and has been widely used for obesity research [32–34]. Sirt1 loss from adipose tissue leads to obesity and metabolic dysfunction [35]. In addition, a Pdpk1-deficient mouse model has demonstrated the importance of Pdpk1 in pancreatic cell mass and glucose homeostasis [36]. Phenotypes associated with diabetes and obesity were not detected for Sin3a and Sqstm1.

PageRank score calculation for user-specific networks: Users can prioritize genes according to human disease associations generated from their own in-house data, increasing their understanding of the genes most affected by their own networks and/or selecting candidate genes for network behavior (Fig. 3b).

## Discussion

We have constructed a gene-phenotype prediction system, TarGo, with a gene interaction network and disease signatures. We found that many results predicted by TarGo matched the preliminary IMPC phenotyping data. TarGo can, therefore, be used to suggest candidate target genes for research using human disease models.

## Conclusion

This new data will be extremely valuable for understanding human diseases and related phenotypes. It is crucial that the large volumes of data are regularly updated, as they are sourced from many biological databases.

## Materials and methods

### Gene-phenotype association by the TSPR score

Using the TSPR algorithm alongside the hyperlink matrix and weight matrix as populated above, we predict gene-phenotype association rankings. We construct a two-part pipeline. The first part was the calculation of gene-disease (or phenotype) rank with TSPR, and the second part semi-automatically separated out useful genes using TrustRank.

The TSPR score was calculated as follows:

$$ TSPR=\left(\mathbf{d}\right)\ \mathit{\Pr}+\left(\mathbf{1}-\mathbf{d}\right) pa\kern0.84em pa=\left\{\begin{array}{l}\frac{1}{t_j},i\in {t}_j\\ {}o,i\notin {t}_j\end{array}\right\} $$

The dumping factor, d = 0.85, is the probability that a node would move to a different node [37]. Pr is the sum of hyperlink matrix and dangling matrix. t_j_ is the set of annotated genes in phenotype j. If gene i is annotated in phenotype j, the value of weight matrix pa is 1/|t_j_|, and |t_j_| was the number of annotated genes in phenotype j. If gene i is not annotated in phenotype j, the value of pa is 0. The weight matrix p introduced bias in all iterations of the TSPR computation. The TSPR score/matrix is the sum of all contributions made by nodes linking to it.

We applied TrustRank to decrease false positives in the prediction result. Gyöngyi and researchers proposed TrustRank to separate useful webpages from spam [14]. Using manual identification of reputable pages, this method identified other pages that were likely to be trustworthy based on their connectivity with the reputable pages. Highly related genes for a particular phenotype are normalized using the TrustRank score [14]. The TrustRank score is calculated as follows:

$$ \mathrm{TrustRank}=\mathrm{d}\times \Pr \times \mathrm{t}+\left(1\hbox{-} \mathrm{d}\right)\times \mathrm{gd}\left(\mathbf{t}=\mathbf{TrustRank}\ \mathbf{score}\ \mathbf{vector}\right). $$

The vector for good seeds, gd, represented the number of highly ranked genes annotated for a particular phenotype. We selected seed genes that ranked highly on their TSPR score. In this study, we selected the top 3000 seed genes. The seed gene, i, is a defined signature gene for a particular phenotype. If gene i is a good seed gene for that phenotype, then the value of the non-uniform vector gd would be 1/|gd|, where |gd| is the number of good seed genes. If gene i was not a good seed, then the value would be 0.

We predicted the association for gene-phenotype using both the TSPR and TrustRank algorithms. A high-ranked gene in the prediction result is highly related with a particular phenotype and closely interacted with signature genes. To calculate the association for gene-phenotype, we selected phenotypes that interacted with more than 10 signature genes.

Using Spammass, we measure the impact of good seed links on the ranking. The Spammass score is used as an indicator of reliability or confidence for the gene ranking scores. The Spammass score is calculated as 1-(TrustRank/TSPR). If the Spammass score is < 0, the gene’s rank is elevated by good seeds. If the Spammass score is > 0, the gene rank is elevated by an unrelated gene.

## Supplementary information

**Additional file 1: Figure S1.** Correlation distribution between node degree and rank score. **Figure S2.** Sensitivity and specificity for MP and MeSH terms. **Figure S3.** Sensitivity for network and ontology method. **Figure S4.** AUC values for different dumping factors. X axis is dumping factor and Y axis is AUC value. To calculate AUC across different dumping factor, we selected the top 10% genes from the prediction result. ROC analysis was performed using the R ROCR package.

## Data Availability

http://combio.snu.ac.kr/targo

## Abbreviations

ENU: N-ethyl-N-nitrosourea
GEM: genetically engineered mouse
IMPC: International Mouse Phenotyping Consortium
KO: knockout
MeSH: Medical Subject Headings
MGI: Mouse Genome Informatics
MP: Mammalian Phenotype
PPIs: protein-protein interactions
ROC: Receiver operating characteristic
TarGo: Target gene selection system for Genetically engineered mouse models
TSPR: Topic-Sensitive PageRank

## References

[1] Koscielny G, Yaikhom G, Iyer V, Meehan TF, Morgan H, Atienza-Herrero J, Blake A, Chen CK, Easty R, Di Fenza A, Fiegel T, Grifiths M, Horne A, Karp NA, Kurbatova N, Mason JC, Matthews P, Oakley DJ, Qazi A, Regnart J, Retha A, Santos LA, Sneddon DJ, Warren J, Westerberg H, Wilson RJ, Melvin DG, Smedley D, Brown SD, Flicek P, Skarnes WC, Mallon AM, Parkinson H. The International Mouse Phenotyping Consortium Web Portal, a unified point of access for knockout mice and related phenotyping data. Nucleic Acids Res 2013. 10.1093/nar/gkt977 PMC3964955 24194600

[2] Ala U, Piro RM, Grassi E, Damasco C, Silengo L, Oti M, Provero P, Di Cunto F (2008). Prediction of human disease genes by human-mouse conserved coexpression analysis. PLoS Comput Biol.

[3] Guan Y, Gorenshteyn D, Burmeister M, Wong AK, Schimenti JC, Handel MA, Bult CJ, Hibbs MA, Troyanskaya OG (2012). Tissue-specific functional networks for prioritizing phenotype and disease genes. PLoS Comput Biol.

[4] Singh-Blom UM, Natarajan N, Tewari A, Woods JO, Dhillon IS, Marcotte EM (2013). Prediction and validation of gene-disease associations using methods inspired by social network analyses. PLoS One.

[5] Robinson PN, Mundlos S (2010). The human phenotype ontology. Clin Genet.

[6] Smith CL, Eppig JT (2009). The mammalian phenotype ontology: enabling robust annotation and comparative analysis. Wiley Interdiscip Rev Syst Biol Med.

[7] Groth P, Pavlova N, Kalev I, Tonov S, Georgiev G, Pohlenz HD, Weiss B (2007). PhenomicDB: a new cross-species genotype/phenotype resource. Nucleic Acids Res.

[8] Sardana Divya, Vasa Suresh, Vepachedu Nishanth, Chen Jing, Gudivada Ranga Chandra, Aronow Bruce J., Jegga Anil G. (2010). PhenoHM: human–mouse comparative phenome–genome server. Nucleic Acids Research.

[9] Chen CK, Mungall CJ, Gkoutos GV, Doelken SC, Kohler S, Ruef BJ, Smith C, Westerfield M, Robinson PN, Lewis SE, Schofield PN, Smedley D (2012). MouseFinder: candidate disease genes from mouse phenotype data. Hum Mutat.

[10] Hoehndorf R, Schofield PN, Gkoutos GV (2011). PhenomeNET: a whole-phenome approach to disease gene discovery. Nucleic Acids Res.

[11] Smedley D, Oellrich A, Kohler S, Ruef B, Sanger Mouse Genetics P, Westerfield M, Robinson P, Lewis S, Mungall C (2013). PhenoDigm: analyzing curated annotations to associate animal models with human diseases. Database (Oxford).

[12] Smedley D, Kohler S, Czeschik JC, Amberger J, Bocchini C, Hamosh A, Veldboer J, Zemojtel T, Robinson PN (2014). Walking the interactome for candidate prioritization in exome sequencing studies of Mendelian diseases. Bioinformatics.

[13] Hwang T, Atluri G, Xie M, Dey S, Hong C, Kumar V, Kuang R (2012). Co-clustering phenome-genome for phenotype classification and disease gene discovery. Nucleic Acids Res.

[14] Gyöngyi Z, Garcia-Molina H, Pedersen J (2004). Combating Web Spam with TrustRank. Proceedings of the International Conference on Very Large Data Bases.

[15] Beck T, Hastings RK, Gollapudi S, Free RC, Brookes AJ (2014). GWAS central: a comprehensive resource for the comparison and interrogation of genome-wide association studies. Eur J Hum Genet.

[16] Köhler Sebastian, Doelken Sandra C., Mungall Christopher J., Bauer Sebastian, Firth Helen V., Bailleul-Forestier Isabelle, Black Graeme C. M., Brown Danielle L., Brudno Michael, Campbell Jennifer, FitzPatrick David R., Eppig Janan T., Jackson Andrew P., Freson Kathleen, Girdea Marta, Helbig Ingo, Hurst Jane A., Jähn Johanna, Jackson Laird G., Kelly Anne M., Ledbetter David H., Mansour Sahar, Martin Christa L., Moss Celia, Mumford Andrew, Ouwehand Willem H., Park Soo-Mi, Riggs Erin Rooney, Scott Richard H., Sisodiya Sanjay, Vooren Steven Van, Wapner Ronald J., Wilkie Andrew O. M., Wright Caroline F., Vulto-van Silfhout Anneke T., Leeuw Nicole de, de Vries Bert B. A., Washingthon Nicole L., Smith Cynthia L., Westerfield Monte, Schofield Paul, Ruef Barbara J., Gkoutos Georgios V., Haendel Melissa, Smedley Damian, Lewis Suzanna E., Robinson Peter N. (2013). The Human Phenotype Ontology project: linking molecular biology and disease through phenotype data. Nucleic Acids Research.

[17] Rogers FB (1963). Medical subject headings. Bull Med Libr Assoc.

[18] Weinreich SS, Mangon R, Sikkens JJ, Teeuw ME, Cornel MC (2008). Orphanet: a European database for rare diseases. Ned Tijdschr Geneeskd.

[19] Jimeno-Yepes AJ, Sticco JC, Mork JG, Aronson AR (2013). GeneRIF indexing: sentence selection based on machine learning. BMC Bioinformatics.

[20] Koike A, Kobayashi Y, Takagi T (2003). Kinase pathway database: an integrated protein-kinase and NLP-based protein-interaction resource. Genome Res.

[21] Lee I, Blom UM, Wang PI, Shim JE, Marcotte EM (2011). Prioritizing candidate disease genes by network-based boosting of genome-wide association data. Genome Res.

[22] Ramos EM, Hoffman D, Junkins HA, Maglott D, Phan L, Sherry ST, Feolo M, Hindorff LA (2014). Phenotype-genotype integrator (PheGenI): synthesizing genome-wide association study (GWAS) data with existing genomic resources. Eur J Hum Genet.

[23] Qiu F, Xu Y, Li K, Li Z, Liu Y, DuanMu H, Zhang S, Li Z, Chang Z, Zhou Y, Zhang R, Zhang S, Li C, Zhang Y, Liu M, Li X (2012). CNVD: text mining-based copy number variation in disease database. Hum Mutat.

[24] He Y, Zhang M, Ju Y, Yu Z, Lv D, Sun H, Yuan W, He F, Zhang J, Li H, Li J, Wang-Sattler R, Li Y, Zhang G, Xie L (2012). dbDEPC 2.0: updated database of differentially expressed proteins in human cancers. Nucleic Acids Res.

[25] Blake JA, Bult CJ, Eppig JT, Kadin JA, Richardson JE (2014). Mouse genome database G. the mouse genome database: integration of and access to knowledge about the laboratory mouse. Nucleic Acids Res.

[26] Sing T, Sander O, Beerenwinkel N, Lengauer T (2005). ROCR: visualizing classifier performance in R. Bioinformatics.

[27] Donovan A, Lima CA, Pinkus JL, Pinkus GS, Zon LI, Robine S, Andrews NC (2005). The iron exporter ferroportin/Slc40a1 is essential for iron homeostasis. Cell Metab.

[28] Zheng J, Lu Z, Kocabas F, Bottcher RT, Costell M, Kang X, Liu X, Deberardinis RJ, Wang Q, Chen GQ, Sadek H, Zhang CC (2014). Profilin 1 is essential for retention and metabolism of mouse hematopoietic stem cells in bone marrow. Blood.

[29] Graham BH, Waymire KG, Cottrell B, Trounce IA, MacGregor GR, Wallace DC (1997). A mouse model for mitochondrial myopathy and cardiomyopathy resulting from a deficiency in the heart/muscle isoform of the adenine nucleotide translocator. Nat Genet.

[30] Skarnes WC, Rosen B, West AP, Koutsourakis M, Bushell W, Iyer V, Mujica AO, Thomas M, Harrow J, Cox T, Jackson D, Severin J, Biggs P, Fu J, Nefedov M, de Jong PJ, Stewart AF, Bradley A (2011). A conditional knockout resource for the genome-wide study of mouse gene function. Nature.

[31] Lloyd DJ, Wheeler MC, Gekakis N (2010). A point mutation in Sec61alpha1 leads to diabetes and hepatosteatosis in mice. Diabetes.

[32] Mingrone G, Manco M, Iaconelli A, Gniuli D, Bracaglia R, Leccesi L, Calvani M, Nolfe G, Basu S, Berria R (2008). Prolactin and insulin ultradian secretion and adipose tissue lipoprotein lipase expression in severely obese women after bariatric surgery. Obesity (Silver Spring).

[33] Sanchez J, Priego T, Pico C, Ahrens W, De Henauw S, Fraterman A, Marild S, Molnar D, Moreno LA, Peplies J, Russo P, Siani A, Tornaritis M, Veidebaum T, Palou A, Consortium I (2012). Blood cells as a source of transcriptional biomarkers of childhood obesity and its related metabolic alterations: results of the IDEFICS study. J Clin Endocrinol Metab.

[34] Zhou L, Zhang J, Fang Q, Liu M, Liu X, Jia W, Dong LQ, Liu F (2009). Autophagy-mediated insulin receptor down-regulation contributes to endoplasmic reticulum stress-induced insulin resistance. Mol Pharmacol.

[35] Chalkiadaki A, Guarente L (2012). High-fat diet triggers inflammation-induced cleavage of SIRT1 in adipose tissue to promote metabolic dysfunction. Cell Metab.

[36] Hashimoto N, Kido Y, Uchida T, Asahara S, Shigeyama Y, Matsuda T, Takeda A, Tsuchihashi D, Nishizawa A, Ogawa W, Fujimoto Y, Okamura H, Arden KC, Herrera PL, Noda T, Kasuga M (2006). Ablation of PDK1 in pancreatic beta cells induces diabetes as a result of loss of beta cell mass. Nat Genet.

[37] Haveliwala TH (2003). Topic-sensitive PageRank: a context-sensitive ranking algorithm for web search. IEEE Trans Knowl Data Eng.

