# Supplementary material for: TarGo: network based target gene selection system for human disease related mouse models
Source: Lab Anim Res. 2019 Nov 13;35:23. doi: 10.1186/s42826-019-0023-z (PMC7081697; doi:10.1186/s42826-019-0023-z)
Supplement: Supplementary file 1 — Additional file 1: Figure S1. Correlation distribution between node degree and rank score. Figure S2. Sensitivity and specificity for MP and MeSH terms. Figure S3. Sensitivity for network and ontology method. Figure S4. AUC values for different dumping factors. X axis is dumping factor and Y axis is AUC value. To calculate AUC across different dumping factor, we selected the top 10% genes from the prediction result. ROC analysis was performed using the R ROCR package. [file 42826_2019_23_MOESM1_ESM.docx]

## Hub node effect in ranking

A hub node in a network is defined as a highly connected node, and is always highly ranked in network topological analysis. TrustRank score is calculated using the number of hyperlinks. **A hub node is highly probable to be a false positive due to the high propagation power in the network**. We examined the effect for a hub node in the TrustRank score. Using Pearson method, we examined the association between TrustRank score and node degree. The correlation between node degree and TrustRank score was calculated for each MP term or MeSH term. If phenotypes show high correlation, the hub node is highly ranked in the prediction result. For 98% of MP terms, the correlations between node degree and TrustRank score was smaller than 0.6, while 90% of MeSH terms had a correlation coefficient lower than 0.6. In most phenotypes, the hub node was not highly ranked (Supplement Fig 1). This indicates TarGo is not biased towards highly connected genes with phenotypes.

However some phenotypes showed a high correlation with ranking score (Supplementary figure 3). We assumed that some signature genes are also hub nodes. We divided signature genes into high and low correlation group (high > 0.6, low < 0.6). Using the T test method, we confirmed different node degree values between these high and low correlation groups (p value: 2.293e-13 (MP term), 3.167e-18 (MeSH term)). The high correlation group show a higher node degree than the low correlation group. This means that some high degree genes (e.g. hub genes) are selected by signature genes for phenotypes and can therefore affect the TrustRank score. Even if a small number of phenotypes show a high correlation between node degree and TrustRank score, the hub node is annotated for the phenotype and may be displayed as a true positive in the analysis. This indicates that the TrustRank score is not related to node degree. We used same network topology in the gene ranking calculation, and the association score was calculated so that it was dependent upon other topics, not network topology.


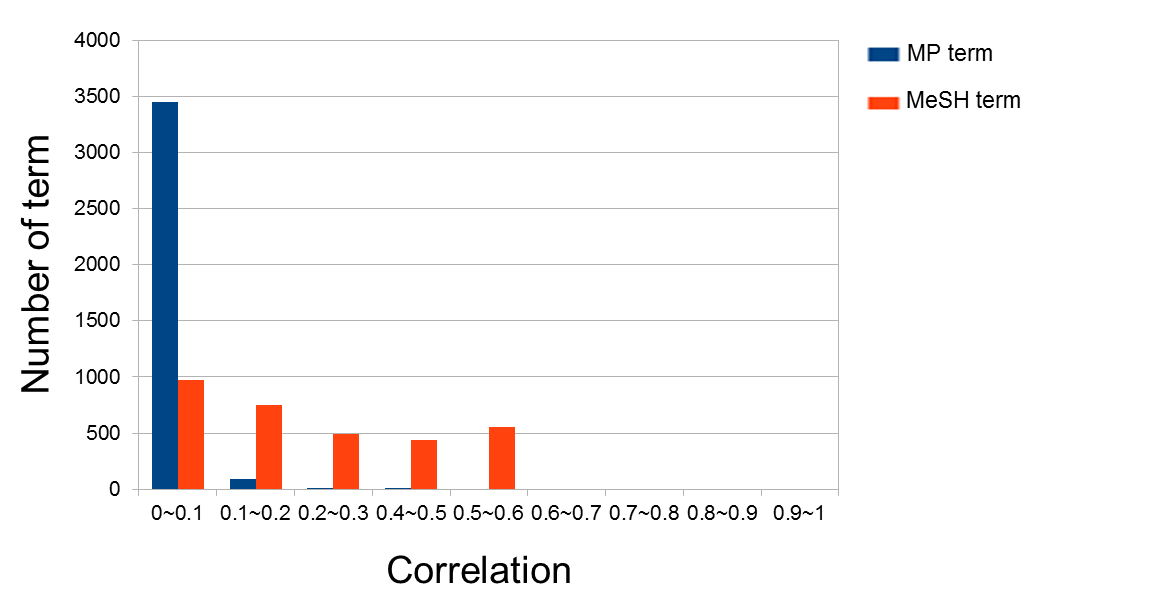


Additional file 1: Figure S1. Correlation distribution between node degree and rank score.

**1.2 GEM target gene prediction with Fisher exact test**

TarGo shows the highly ranked genes for a specific user-selected phenotype using the Fisher exact test in conjunction with top 100 genes in the TrustRank algorithm, allowing the user to select the target gene for their GEM model (Fig 3a). If we know disease related phenotypes and some gene are highly ranked in multiple phenotypes, this gene can be highly associated with selected diseases or phenotypes. TarGo proposed candidate genes in user interesting phenotypes, using Fisher Exact test which were widely used in function prediction, like Gene Set Analysis. We tested the null hypothesis that given gene X in selected MP terms and other MP terms is equally highly ranked. The small P value is greater evidence for rejecting the null hypothesis. So here, the evidence is strong that given gene X in selected MP terms and other MP terms is not equally highly ranked. This approach can be used to indicate candidate genes for phenotype driven screens where the causative gene is unknown.

1.3 **Sensitivity and Specificity**


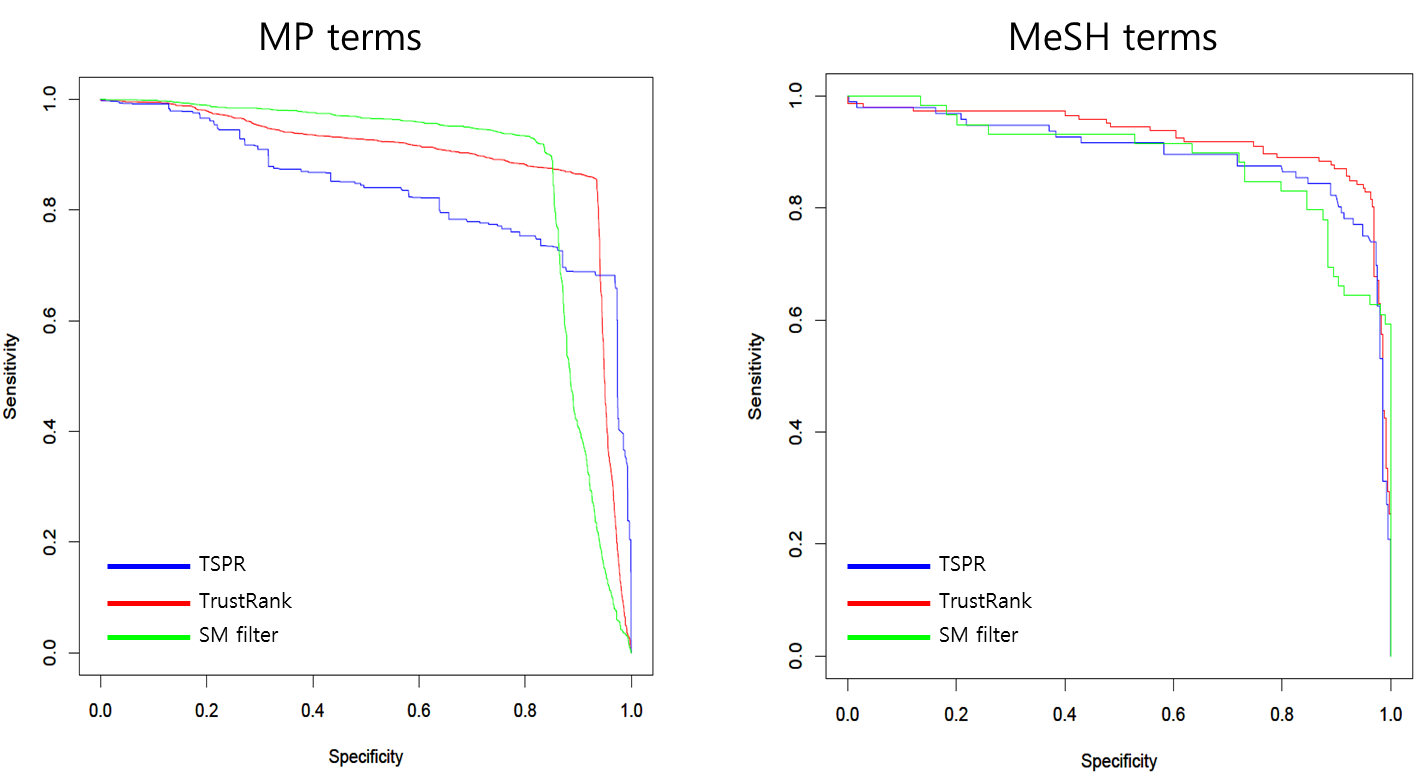


Figure S2. Sensitivity and specificity for MP and MeSH terms

1.4 **Validation of system**

To validate prediction system, we measured sensitivity for network and ontology base method. Phenodigm selected for ontology base method and Protein-Protein interaction, Pathway Common, Human Net were selected for network. All genes (test set) were sorted by high prediction score and measured overlapping with OMIM data set (true set). In top 10% gene in each selected method, ontology base method showed lower sensitivity than network method. From top 40% gene in each selected method, sensitivity was not different between methods.


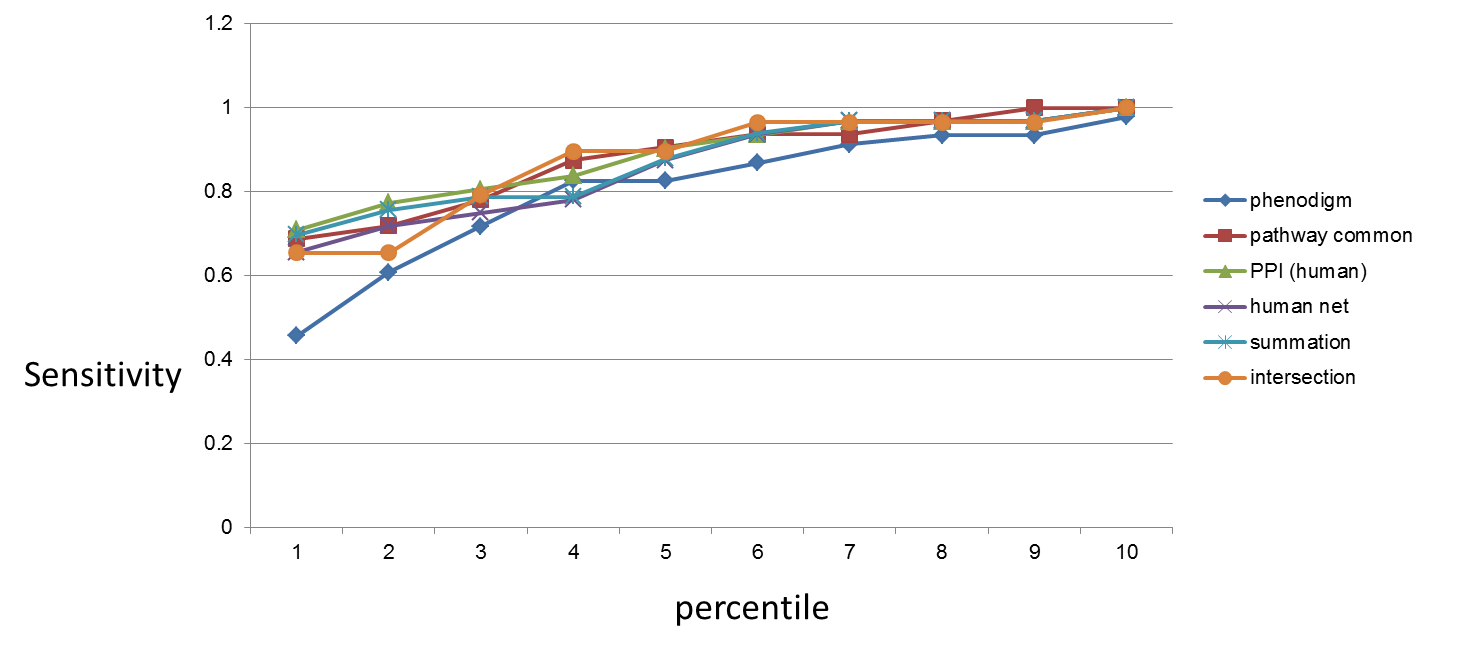


Figure S3. Sensitivity for network and ontology method.

**Figure S4. AUC values for different dumping factors. X axis is dumping factor and Y axis is AUC value. To calculate AUC across different dumping factor, we selected the top 10% genes from the prediction result. ROC analysis was performed using the R ROCR package.**
